# Clinical parameters associated with myocardial hemorrhage in reperfused acute myocardial infarction — a cardiovascular MR study

**DOI:** 10.1186/1532-429X-15-S1-P222

**Published:** 2013-01-30

**Authors:** Yoko Mikami, Andreas Kumar, Tak S Fung, Andrew G Howarth, Matthias G Friedrich

**Affiliations:** 1 Stephenson CMR centre, Calgary, AB, Canada; 2 Laval University, The institute of Cardiology and Pulmonology of Quebec, Quebec, QC, Canada; 3 Information Technologies, University of Calgary, Calgary, AB, Canada; 4 Cardiology, Université de Montréal, Montreal, QC, Canada; 5 Cardiac Sciences, University of Calgary, Calgary, AB, Canada

## Background

Late reperfusion of acutely ischemic myocardium may result in intramyocardial hemorrhage that has been described as a contributor to reperfusion injury. In patients with reperfused acute myocardial infarction (MI), the presence of myocardial hemorrhage can be detected by T2*-weighted cardiovascular magnetic resonance (CMR). This study aimed to identify clinical parameters associated with myocardial hemorrhage.

## Methods

Thirty patients underwent CMR three days after acute reperfused MI including cine, hemorrhage-sensitive T2*-weighted imaging, early (2-5 minutes post contrast) and late gadolinium enhancement (LGE) imaging for microvascular obstruction (MO) and necrosis, respectively. The extent of MO was measured on both early and late post-contrast images. Stepwise logistic regression analysis was performed to identify associations of clinical parameters with the presence of hemorrhage. The parameters tested were age, gender, risk factors, left ventricular (LV) volume and function, the extent of MO on early post-contrast images, the extent of MO on LGE images, the extent of infarction, time from the onset of pain to reperfusion, culprit artery, blood pressure, heart rate, TIMI flow, use of a thrombolytic agent, use of thrombo-aspiration devices, maximum value of troponin T and creatine kinase (CK), LDL, and HbA1c. LV volume was indexed by body surface area and infarct mass and MO mass were indexed by LV myocardial mass.

## Results

Out of 30 patients, 19 patients showed MO and 18 of this group also showed hemorrhage. Indexed LV end-diastolic volume, the indexed MO mass on LGE images, the indexed infarction mass, maximum CK and troponin T were related to the presence of hemorrhage. The indexed infarct mass was the strongest predictor of the presence of hemorrhage (estimated regression coefficient 0.241(SE .095), p=0.011).

## Conclusions

The presence of hemorrhage is associated with larger LV end-diastolic volume, larger infarct size, greater MO on LGE images, higher CK and troponin T value with infarct mass being the strongest predictor. These findings indicate that myocardial hemorrhage is strongly associated with the extent of myocardial damage in reperfused acute MI. However, this study was limited by the small sample size. Future studies with larger sample size could provide more definitive conclusions.

## Funding

This study was supported by a research grant from Canadian Institutes of Health Research, Pfizer Canada Inc. and the Calgary Health Trust.

